# Determinants of hospitalization in Chinese patients with type 2 diabetes receiving a peer support intervention and JADE integrated care: the PEARL randomised controlled trial

**DOI:** 10.1186/s40842-018-0055-6

**Published:** 2018-03-07

**Authors:** Roseanne O. Yeung, Jing-Heng Cai, Yuying Zhang, Andrea O. Luk, Jun-Hao Pan, Junmei Yin, Risa Ozaki, Alice P. S. Kong, Ronald Ma, Wing-Yee So, Chiu Chi Tsang, K. P. Lau, Edwin Fisher, Williams Goggins, Brian Oldenburg, Julianna Chan

**Affiliations:** 1 Division of Endocrinology and Metabolism, Department of Medicine, University of Alberta, 9-111K Clinical Science Building, 11350 83 Avenue, Edmonton, AB T6G 2G3 Canada; 2 Sun Yat-Sen University, Guangzhou, China; 3 Chinese University of Hong Kong, Hong Kong, Hong Kong; 4 Alice Ho Miu Ling Nethersole Hospital, Hong Kong, Hong Kong; 5 North District Hospital, Sheung Shui, Hong Kong; 6 University of North Carolina at Chapel Hill, Chapel Hill, USA; 7 University of Melbourne, Melbourne, Australia; 8 Asia Diabetes Foundation, Hong Kong, Hong Kong

**Keywords:** Structural equation modelling, Quality improvement, Peer support, Integrated care, Hospitalizations, Negative emotions, Adherence

## Abstract

**Background:**

In a randomized controlled trial of 628 Chinese patients with type 2 diabetes receiving multidisciplinary care in the Joint Asia Diabetes Evaluation (JADE) Progam, 372 were randomized to receive additional telephone-based peer support (Peer Empowerment And Remote communication Linked by information technology, PEARL) intervention. After 12 months, all-cause hospitalization was reduced by half in the PEARL group especially in those with high Depression Anxiety and Stress Scale (DASS) scores.

**Methods:**

We used stratified analyses, negative binomial regression, and structural equation modelling (SEM) to examine the inter-relationships between emotions, self-management, cardiometabolic risk factors, and hospitalization.

**Results:**

Hospitalized patients were older, more likely to have heart or kidney disease, and negative emotions than those without hospitalization. Patients with high DASS score who did not receive peer support had the highest hospitalization rates. After adjustment for confounders, peer support reduced the frequency of hospitalizations by 48% with a relative risk of 0.52 (95% CI 0·35–0·79;*p* = 0·0018). Using SEM, improvement of negative emotions reduced treatment nonadherence (Est = 0.240, *p* = 0.034) and hospitalizations (Est=−0.218, *p* = 0.001). The latter was also reduced by an interactive term of peer support and chronic kidney disease (Est = 0.833, *p* = < 0.001) and that of peer support and heart disease (Est = 0.455, *p* = 0.001).

**Conclusions:**

In type 2 diabetes, improvement of negative emotions and peer support reduced hospitalizations, especially in those with comorbidities, in part mediated through improving treatment nonadherence. Integrating peer support is feasible and adds value to multidisciplinary care, augmented by information technology, especially in patients with comorbidities.

**Trial registration:**

NCT00950716 Registered July 31, 2009.

## Background

In clinical trial settings, diabetes complications are preventable with modifiable risk factor control. Despite recent advances in therapeutics, many patients have suboptimal control of cardiometabolic risk factors. In the United States, less than half of the patients have attained recommended goals for diabetes care [1]. In some surveys, patients with type 2 diabetes had 30% increased risk of hospitalization [2] and 50% increased inpatient mortality rate compared to those without diabetes [3]. The co-occurrence of comorbidities, particularly poor mental health, also increases the risk of health care utilization in people with diabetes [4, 5].

One of the greatest challenges in diabetes management is to motivate, initiate, and sustain behavioural changes to maintain metabolic control [6]. While frequent contacts with health care providers (HCP) improves risk factor control [7], peer support has the potential to promote self-management and care quality, especially in low resource settings [8, 9]. “Peer support” is defined as “support from a person who has experiential knowledge of a specific behaviour or stressor and similar characteristics as the target population” [8]. Herein, peer support can empower patients through building trust from shared experiences and by providing informational and emotional support [9].

In type 2 diabetes, peer support has been shown to improve clinical, behavioural, and psychological outcomes [10, 11]. Peer support has also been shown to reduce health care utilisation and cost, although results have not always been consistent [12, 13]. In 2007, we launched a nurse-coordinated diabetes care program augmented by the web-based Joint Asia Diabetes Evaluation Program (JADE Program) [14]. We randomized 628 Chinese patients with type 2 diabetes receiving JADE care to peer support versus JADE alone to evaluate whether a telephone-based peer support program (Peer Empowerment And Remote communication Linked by information technology, PEARL program) would further improve risk factor control and all-cause hospitalizations [15]. At the end of 12 months, patients in both arms had similar improvements in glycemic control and cardiometabolic risk factors. However, the addition of PEARL peer support intervention reduced hospitalization rate compared to JADE care alone, especially in patients with the highest levels of negative emotion as measured by the Depression, Anxiety, and Stress Scale (DASS-21) with a relative risk (RR) of 0.15 (0.07–0.34, *p* < 0.0001). In this post-hoc analysis, we performed detailed analysis to investigate the inter-relationships between emotions, medication adherence, peer support, and hospitalizations.

## Methods

### Study design and hypothesis

The PEARL study was a 12-month randomized controlled trial evaluating the effect of peer support on metabolic control and hospitalizations from 2009 to 2010. The primary results of the PEARL study were published with detailed explanation of methods [15]. Our overarching hypothesis is that a telephone-based peer support intervention may improve negative emotions and medication adherence, thereby improving risk factor control and reducing all-cause hospitalization rates. To further explore the trial findings, we performed the following post-hoc analyses:

1. Univariate analyses of clinical profiles stratified by hospitalization status (ever hospitalized versus never hospitalized)
2. Univariate analyses of clinical profiles stratified by the median levels of negative emotions as measured by the Depression Anxiety Stress Scale (DASS-21) [15].
3. Kaplan Meier and negative binomial regression analyses to evaluate the impact of peer support and negative emotions (DASS-21) on hospitalizations.
4. Structural equation modelling (SEM), which provides a multivariate lens while addressing confounding by multicollinearity, to identify independent key factors leading to hospitalization.

### Ethics, consent, and permissions

This study was approved by the Joint Chinese University of Hong Kong – New Territories East Cluster (CUHK-NTEC) Clinical Research Ethics Committee. All willing patients gave written informed consent and completed validated questionnaires for evaluation of psychological-behavioural measures at baseline and at the end of the study, and were randomized to the PEARL peer support intervention within the JADE program or the JADE program alone in a 1:1 ratio.

### Setting

Hong Kong has a subsidised public healthcare system with the Hospital Authority (HA) providing the bulk of chronic care through its hospitals and clinics which share a unified electronic medical record known as the Clinical Management System (CMS). Based on a series of epidemiological and interventional studies, the JADE Program was established in 2007 to deliver technologically-enhanced and integrated multidisciplinary care through the use of structured data collection, risk stratification, and evidence-based decision support [14].

This 12-month randomized study was conducted in three HA diabetes centres where the JADE Program was implemented. Consecutive patients were recruited throughout the year and randomized as they consented. The JADE Program included an initial 4-h nurse-led assessment including history taking, blood/urine collection, and eye/ft examination where the data were entered into the online portal which electronically generated personalised reports regarding the patient’s risk factors, complications, and risk categories based on findings from the Hong Kong Diabetes Register [14]. Frequency of follow-up visits was recommended based on individualized risk assessment. Chronic kidney disease (CKD) was defined as estimated glomerular filtration rate (eGFR) < 60 ml/min/1.73 m^2^ or dialysis-dependence. Heart disease was defined as a history of myocardial infarction, coronary revascularization, or heart failure.

### The PEARL intervention

#### Peer supporters

The details of the training program for peer supporters had been reported [15]. In brief, 33 motivated patients with HbA1c less than 8% (64 mmol/mol) who had completed a ‘Train the Trainer’ program conducted during four 8-h workshops agreed to be peer supporters. The program focused on mindset, empathic listening, questioning skills, and counselling skills regarding lifestyle modification. Eligibility included Chinese adult patients with type 2 diabetes (18–70 years) who underwent the JADE comprehensive assessment. Exclusions included those in the lowest JADE Risk Category (no diabetes complications, ≤1 risk factor, and low-risk score) [15] or those with suicidal ideation. Each peer supporter was introduced to 10 peers during a 2-h session by nurses and was expected to contact each peer at least 12 times over 12 months in a phone or face-to-face meeting. Conversations or meetings were guided by a script, which addressed diet, exercise, self-monitoring of blood glucose, sick day management, foot care, emotional support, resources for information, and clinical care. Peer supporters were asked to record the duration of each call and any relevant details for the call. The doctors, nurses, and project coordinators met all peer supporters on three occasions for a half-day meeting to share experiences. Confidentiality of patient information was emphasised at each meeting.

### Peers

All Chinese patients with type 2 diabetes aged 18 to 70 years who underwent JADE comprehensive assessments who were receptive were eligible. Exclusion criteria included illiteracy, inability to communicate in Chinese, those in JADE Risk Category 1 (no complications, ≤1 risk factor, and low-risk score), and those receiving psychiatric treatment.

#### Main outcomes and measures

The CMS captured all hospitalizations within the Hong Kong public health care system, classified by the International Classification of Diseases, Ninth Revision (ICD-9) codes on discharge. Measures included the total number of all-cause hospitalizations for each group, frequency of hospitalization per patient, and the total length of stay (TLOS) per patient admission, defined as a count of day admissions plus nights spent in a hospital. Measurements were censored using the CMS on the day of repeat assessment or after 12 months in those who did not return. All patients completed validated questionnaires for psychometric measures including the 9-item Patient Health Questionnaire (PHQ-9) for depressive symptoms [16], the 21-item Depression Anxiety Stress Scale (DASS-21) with sub scores for depression, anxiety and stress [17], the 15-item Chinese Diabetes Distress Scale (CDDS-15) for diabetes-related distress [18], and the 4-item Morisky Medication Adherence Scale for medication adherence [19].

#### Statistical analysis

For each patient, we censored the hospitalization data at their 1 year reassessment. For patients who did not return at 1 year for assessment, we retrieved their results from the Health Authority CMS, if available. Descriptive analyses were presented as mean ± SD or frequency (%) stratified by hospitalization status and negative emotions. The Pearson χ^2^ test, Fisher exact test, t test, Wilcoxon paired test, and Mann-Whitney test were used for comparisons, as appropriate. Significant negative emotion was defined as the top quintile value of DASS-21 score ≥ 17. We used unadjusted Kaplan Meier “time to event” estimates to illustrate the relationship between peer support and negative emotion on time to first hospitalization. Since the majority of patients were not hospitalized, we used zero-inflated negative binomial regression to estimate the relative risk (RR) for the frequency and TLOS of hospitalizations with 95% confidence interval (CI) adjusted for age, sex, disease duration, and risk category to compare with the unadjusted findings. A *p* < 0.05 (2-tailed) was considered significant. We applied intention-to-treat analysis to all randomized patients using the Statistical Package for Social Sciences (version 20.0, Chicago, USA) for descriptive analysis and Statistical Analysis Software for the negative binomial regression analysis (version 9.3). Missing data were omitted from the analyses.

#### Structural equation modelling (SEM)

**Fig. 1:**
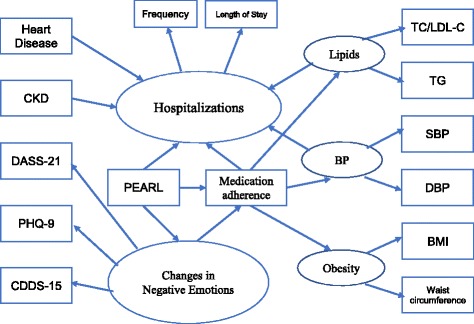
Proposed structural equation model with hypothesized explanatory and outcome variables (rectangle: measured variables; oval: latent variables) and interlinking paths to explain the multi-causality of hospitalization (15). Abbreviations: PEARL: peer support intervention, Frequency: number of hospitalization episodes, Stay: Length of hospital stay, PHQ-9: 9-item Patient Health Questionnaire assessing depressive symptoms, DASS-21: 21-item Depression Anxiety Stress Scale, CDDS-15: 15-item Chinese Diabetes Distress Scale, Medication Adherence: 4-item Morisky Medication Adherence Scale, CKD: Chronic Kidney Disease with eGFR 15–60 ml/min/1.73 m^2^, CVD: Cardiovascular disease including coronary heart disease (myocardial infarction, unstable angina, percutaneous coronary intervention, coronary bypass operation), stroke, peripheral vascular disease (lower extremity amputation, absent foot pulses with ankle:brachial ratio < 0.9 and/or lower limb revascularization), BP: Blood pressure, SBP (systolic) DBP (diastolic), TC/LDL-C: Total cholesterol/LDL-cholesterol, TG: Triglycerides, BMI: Body mass index, Waist: waist circumference, N.B. Baseline explanatory measures were analyzed

We have chosen to use SEM because it allows examination of relationships among observed and latent (unobserved) variables. Moreover, SEM eliminates possible multicollinearity induced by highly correlated indicators and provides simple interpretations among observed and latent variables [20]. We first used factor analysis to group highly correlated measured variables into corresponding latent factors taking measurement errors into account. The structure of our factor loading was determined on subjective, a priori clinical knowledge. These included 1) “hospitalization” (grouped by TLOS and frequency of hospital admissions); 2) “change in negative emotions” from baseline to end of study (grouped by changes in CDDS-15, PHQ-9, and DASS-21); 3) “blood pressure” from systolic and diastolic blood pressure; 4) “lipids” from triglycerides, LDL-C, and total cholesterol; and 5) “obesity” from body mass index (BMI) and waist circumference. We did not include HbA1c due to similar changes between the two groups (PEARL: − 0.30 (− 0.47 to − 0.12) and non-PEARL group: − 0.29 (− 0.47 to − 0.12) at one year [15]. In the SEM model, we included age, gender and peer support (yes/no: 1/0) as determinants for hospitalization based on results from our primary paper [15]. Supported by other studies, we included medication adherence (measured by the 4-item Morisky Medication Adherence Scale) [21] and diabetes complications [CKD and heart disease coded as 1/0)] known to increase health care utilization [22]. We regressed multiple equations linking peer support to “hospitalization” through “change in negative emotions”, “adherence”, “blood pressure”, “lipids”, and “obesity” factors (Fig. 1). We considered the effects of “change in negative emotions” on medication adherence and tested the interactive terms of (peer support×CKD) and (peer support×heart disease) on “hospitalization”. Mplus software was used to obtain the estimates and goodness-of-fit indices with optimal indexes defined as Chi square test> 0.05, root mean square error of approximation smaller than 0.08 and Comparative Fit Index (CFI) value approximate to 1 [23].

## Results

The study participants’ clinical profiles have been published and were similar between the 2 groups [age:54.7 ± 9.3 years; 57% men; disease duration:9.4 ± 7.7 years; HbA1c: 8.2% ± 1.6% (66 ± (− 6)mmol/mol); systolic BP:136 ± 19 mmHg; LDL-C:2.89 ± 0.82 mmol/L; 17.4% cardiovascular-renal disease; 34.9% insulin-treated] [15]. After a follow-up period of 414 ± 55 days, 144 (22.9%) patients were hospitalized at least once. They were older, more likely to receive multiple medications, and 2–3 times more likely to have CKD and heart disease and had higher DASS-21 scores than those without hospitalization (Table 1). Those with the most negative emotions (DASS-21 ≥ 17, *n* = 124) were more likely to be female, less well-educated, more obese, had more depressive symptoms and poorer medication adherence than those with lower scores.

**Table 1:** Clinical, biochemical, psychological and behavioural parameters stratified by hospitalization and baseline DASS-21 score

	Hospital admission			Baseline DASS-21 score		
	No (*n* = 484)	Yes (*n* = 144)	*P* value	< 17 (*n* = 503)	≥17 (*n* = 124)	*P* value
Age, years	54·1 ± 9·4	56·7 ± 8·6	0·003	54·9 ± 9·1	54·0 ± 9·7	0·358
Male, n(%)	274 (56·6)	81 (56·3)	0·939	295 (58·6)	59 (47·6)	0·026
Diabetes duration, years	9·1 ± 7·7	10·3 ± 7·8	0·122	9·4 ± 7·7	9·5 ± 8·0	0·845
Education						
< 6 years	165 (34·1)	63 (43·8)	0·079	171 (34·0)	57 (46·0)	0·005
6–11 years	247 (51·0)	62 (43·1)		252 (50·1)	56 (45·2)	
> 11 years	72 (14·9)	19 (13·2)		80 (15·9)	11 (8·9)	
Complications						
Chronic kidney disease	25 (5·2)	21 (14·8)	< 0·001	34 (6·8)	12 (9·8)	0·251
Diabetic retinopathy	166 (34·3)	56 (38·9)	0·312	169 (33·6)	53 (42·7)	0·057
All heart events	41 (8·5)	26 (18·3)	0·001	52 (10·4)	15 (12·1)	0·586
JADE Risk categories						
Very high risk	73 (15·1)	36 (25·0)	0·003	86 (17·1)	23 (18·5)	0·474
High risk	385 (79·5)	104 (72·2)		391 (77·7)	97 (78·2)	
Medium risk	24 (5·0)	4 (2·8)		24 (4·8)	4 (3·2)	
Low risk	2 (0·4)	0 (0·0)		2 (0·4)	0 (0·0)	
Treatments						
Insulin	165 (34·1)	54 (37·5)	0·451	174 (34·6)	45 (36·3)	0·722
Oral anti-diabetic drugs	407 (84·1)	127 (88·2)	0·226	423 (84·1)	110 (88·7)	0·197
On BP drugs	305 (63·0)	104 (72·2)	0·042	327 (65·0)	81 (65·3)	0·948
On lipid lowering drugs	194 (40·1)	84 (58·3)	< 0·001	218 (43·3)	59 (47·6)	0·394
Risk factors control						
Body mass index, kg/m^2^	26·8 ± 4·3	27·1 ± 5·0	0·563	26·6 ± 4·3	27·8 ± 4·8	0·009
Systolic blood pressure, mmHg	135·6 ± 18·2	136·4 ± 20·9	0·633	136·2 ± 18·4	134·2 ± 20·7	0·307
Diastolic blood pressure, mmHg	80·2 ± 10·5	79·0 ± 11·2	0·227	80·3 ± 10·5	78·6 ± 11·5	0·115
Hemoglobin A1c, % (mmol/mol)	8.2 ± 1.6 (66.0 ± 17.9)	8.2 ± 1.6 (65.7 ± 17.7)	0.867	8.2 ± 1.6 (65.6 ± 17.7)	8.3 ± 1.7 (67.2 ± 18.7)	0.394
Total cholesterol, mmol/L	4·91 ± 1·07	4·71 ± 1·06	0·044	4·83 ± 1·07	4·99 ± 1·08	0·160
Triglyceride, mmol/L	1·40 (1·00–2·00)	1·49 (1·00–1·97)	0·704	1·40 (1·00–1·97)	1·52 (1·00–2·30)	0·076
HDL-C, mmol/L	1·20 ± 0·36	1·23 ± 0·36	0·433	1·21 ± 0·35	1·17 ± 0·37	0·265
LDL-C, mmol/L	2·92 ± 0·78	2·77 ± 0·91	0·046	2·86 ± 0·79	2·99 ± 0·91	0·131
Estimated GFR, ml/min/1·73m^2^	111·9 ± 33·1	102·6 ± 36·8	0·005	110·2 ± 33·7	107·7 ± 36·1	0·471
Microalbuminuria	127 (26·6)	39 (27·5)	0·843	134 (27·0)	31 (25·6)	0·765
Psychological assessment						
PHQ-9 score	4·1 ± 4·1	4·7 ± 4·2	0·131	3·1 ± 3·1	8·6 ± 4·9	< 0·001
CDDS-15 score	36·8 ± 13·5	37·0 ± 12·2	0·849	34·8 ± 12·8	45·1 ± 11·6	< 0·001
DASS-21 score						
Total	9·3 ± 10·0	14·0 ± 14·0	< 0·001	5·9 ± 4·7	28·7 ± 11·3	< 0·001
DASS-depression	2.75 ± 3.80	4.23 ± 5.02	0.001	1.53 ± 1.85	9.48 ± 4.78	< 0·001
DASS-anxiety	2.71 ± 3.12	4.20 ± 4.63	< 0·001	1.76 ± 1.65	8.33 ± 4.39	< 0·001
DASS-stress	3.79 ± 3.97	5.24 ± 5.02	0.002	2.55 ± 2.47	10.59 ± 4.01	< 0·001
Medication adherence						
High	200 (43·8)	65 (47·8)	0·248	225 (47·3)	40 (34·5)	0·021
Intermediate	228 (49·9)	66 (48·5)		225 (47·3)	68 (58·6)	
Low	29 (6·3)	5 (3·7)		26 (5·5)	8 (6·9)	

Definitions of risk categories

1. Very High-Risk group with clinically evident cardiovascular-renal complications

2. High-Risk group with ≥3 stratification parameters and/or values above the high specificity cut off for any one of the risk scores and/or eGFR< 60 ml/min/1.73 m^2^

3. Medium Risk group with two stratification parameters and values above the high sensitivity cut off but lower than the high specificity cut off for any of the risk scores and eGFR between 60-90 ml/min/1.73 m^2^ and

4. Low-Risk group with one or fewer stratification parameter and values below the high sensitivity cut off for all risk scores and eGFR≥90 ml/min/1.73 m^2^

Definitions of complications and risk parameters:

Chronic kidney disease (CKD): estimated glomerular filtration rate 15–60 ml/min per 1.73 m^2^

Cardiovascular complication: coronary heart disease (myocardial infarction, unstable angina, percutaneous coronary intervention, coronary bypass operation), stroke, peripheral vascular disease (lower extremity amputation, absent foot pulses with ankle:brachial ratio < 0.9 and/or lower limb revascularization)

Renal complications: End stage renal disease (ESRD) requiring dialysis and/or eGFR< 15 ml/min/1.73m^2^

Microalbuminuria: urinary albumin:creatinine ratio (ACR): ≥2.5 mg/mmol (men) and 3.5 mg/mmol (women)

Macroalbuminuria: urinary ACR > 25 mg/mmol

Retinopathy (typical retinal changes including vitrectomy, haemorrhages, and exudates)

Medication adherence: measured by 4-item Morisky Medication Adherence Scale where 4 is regarded as high adherence, score 2–3 as intermediate adherence, and 0–1 as low adherence

**Fig. 2:**
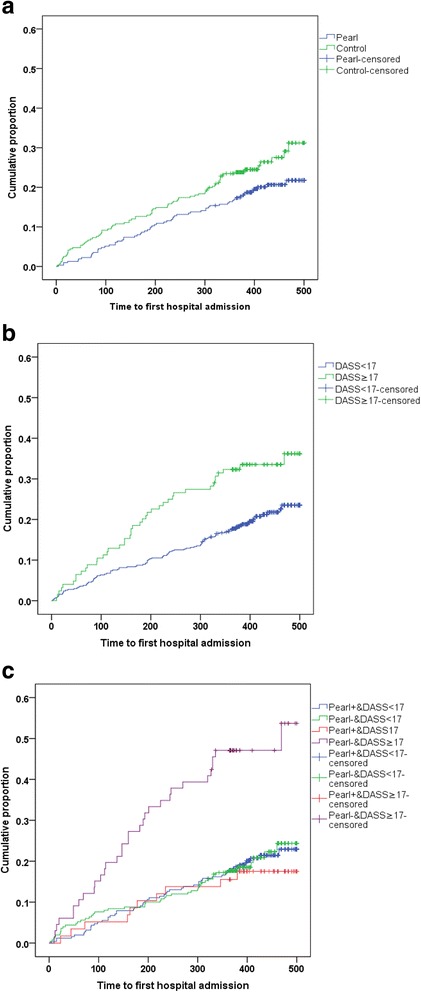
Kaplan Meier curves showing the cumulative proportions of people with type 2 diabetes requiring hospitalization. **a** Stratified by assignment to peer support (PEARL). **b** Stratified by baseline negative emotions (DASS≥17). **c** Stratified by peer support and baseline negative emotions. Pearl denotes peer support intervention. Depression Anxiety Stress Scale are denoted as DASS, using ≥17 points as the cut off for those in the top quintile of subjects with negative emotions

On Kaplan Meier analysis, patients who had DASS-21 ≥ 17 and did not receive peer support had the highest hospitalization rates than the other 3 groups stratified by peer support and DASS-21 scores (Fig. 2). Upon stratification by presence and absence of negative emotions at baseline, peer support tended to improve negative emotions reaching significance for medication adherence in those with the highest levels of negative emotions. Using negative binomial regression, and adjusting for age, gender, disease duration, JADE risk category, and DASS-21 score, peer support reduced hospitalization [RR 0.52 (95% CI 0.35–0.79)] and shortened TLOS [RR 0.46 (95% CI 0.25–0.85)].

### SEM findings

**Fig. 3:**
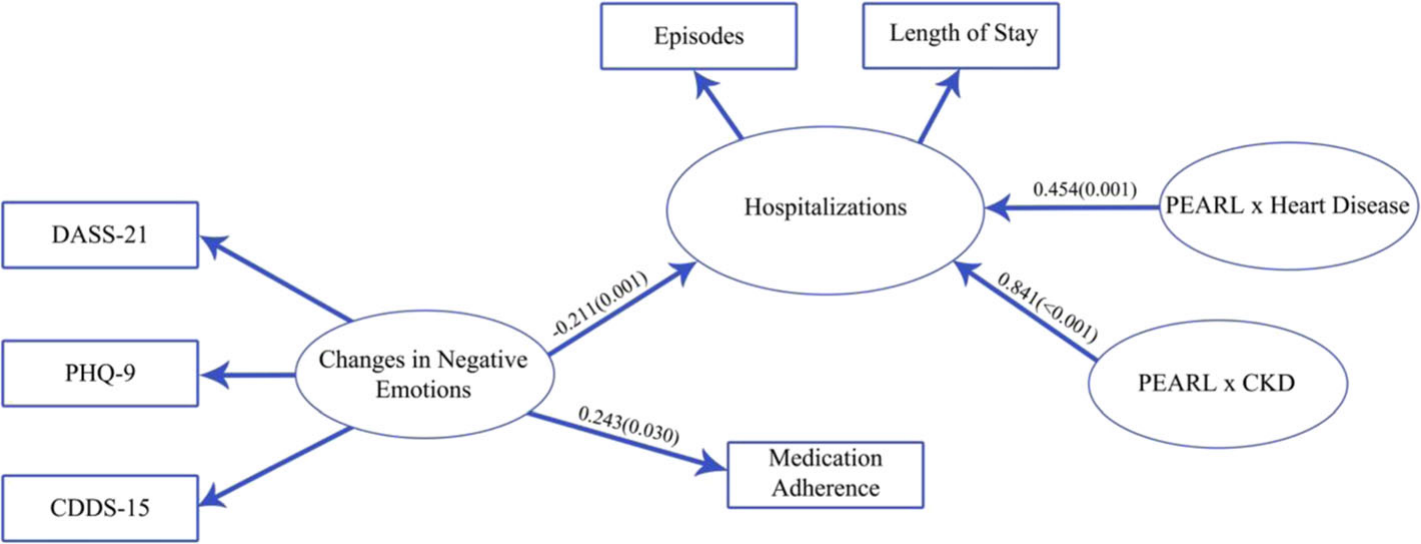
A structural equation model (SEM) showing the effects of change in negative emotions (DASS, PHQ9, CDDS) on hospitalizations (admission episodes and stay) and medication adherence as well as the interaction between peer support and CKD/CVD on hospitalizations. Goodness of fit indices of the model: pvalue of the Chi-squared test is 0.1330; the root mean square error of approximation is 0.019, the CFI value is 0.978. The figure represents the estimates of latent variable relationships to outcome variables with pvalues in parentheses (refer to Table 2 for full details of the SEM). Abbreviations: PHQ-9: 9-item Patient Health Questionnaire assessing depressive symptoms, DASS-21: 21-item Depression Anxiety Stress Scale, CDDS-15: 15-item Chinese Diabetes Distress Scale, Medication Adherence: 4-item Morisky Medication Adherence Scale, CKD: Chronic Kidney Disease as estimated glomerular filtration rate 15–60 ml/min/1.73 m , CVD: Cardiovascular disease including coronary heart disease (myocardial infarction, unstable angina, percutaneous coronary intervention, coronary bypass operation), stroke, peripheral vascular disease (lower extremity amputation, absent foot pulses with ankle:brachial ratio < 0.9 and/or lower limb revascularization)

Based on our original conceptual framework, we formulated a SEM that proposed explanatory pathways leading to hospitalization (Fig. 1). The “blood pressure”, “lipids”, or “obesity” pathways were not significant and thus were excluded from subsequent model building. After fitting data to the different pathways, the final SEM (Fig. 3) demonstrated a significant negative relationship of “change in negative emotions” on “hospitalization” and direct positive effect on “adherence”. In other words, patients with reduced negative emotions were less likely to be hospitalized and were more adherent to medications. Since hospitalized patients were more likely to have CKD, heart disease, and negative emotions, we also tested the interactions between peer support and comorbidities on hospitalization and found strong positive interactions between peer support and CKD, as well as peer support and heart disease on hospitalization. Table 2 quantifies these relationships, which indicate that in patients without CKD, the difference in “hospitalization” values between the PEARL and non-PEARL group was −0.026 compared to 0.807 (−0.026 + 0.833 = 0.807) in those with CKD (*P* = 0.001). For heart disease, the respective values were −0.026 and 0.455 (−0.026 + 0.455 = 0.429) (*P* < 0.001). Our model’s goodness of fit indices revealed a Chi-squared test *p*-value of 0.1330, indicating that our SEM model supported our hypothesis (i.e. not rejected by the data). The SEM model fitting was optimal with the root mean square error of approximation being 0.019, and the CFI value being 0.978, which were better than the predetermined respective criteria for model fitting (less than 0.08 and higher than 0.95).

**Table 2:** Summary of the estimated coefficients of the measurement and structural equation

Measurement equation			
Latent variable	Indicators	Est. (*P* Value)	Standard Error
Change in negative emotions	CDDS	1.000 (ref)	NA (ref)
	PHQ-9	0.874 (< 0.001)	0.137
	DASS-21	1.390 (< 0.001)	0.273
Hospitalisation	Length of stay	1.000 (ref)	NA (ref)
	Frequency of hospitalisation	1.745 (< 0.001)	0.098
Structural equation			
Dependent (outcome) variable	Independent (explanatory) Variable	Est. (*P* Value)	Standard Error
Hospitalisation	Change in negative emotions	−0.218 (0.001)	0.065
	Medication adherence	0.005 (0.833)	0.022
	Age	0.013 (0.552)	0.022
	Peer support	−0.026 (0.576)	0.046
	CKD	0.065 (0.580)	0.118
	Heart disease	−0.001 (0.993)	0.101
	Peer support×CKD	0.833 (< 0.001)	0.171
	Peer support× Heart disease	0.455 (0.001)	0.141
Medication adherence	Change in negative emotions	0.240 (0.034)	0.113
Change in negative emotions	Gender	−0.022 (0.684)	0.054
	Education	0.031 (0.316)	0.031
	Peer Support	0.039 (0.471)	0.053

Goodness of fit indices of the model: *p*-value of the Chi-squared test is 0.1330; the root mean square error of approximation is 0.019, the CFI value is 0.978

*CKD* Chronic kidney disease defined by eGFR< 60 ml/min/1.73m^2^ or dialysis-dependence. Heart disease defined by a history of myocardial infarction, percutaneous coronary intervention, and heart failure

## Discussion

In our primary analysis of this 12-month randomized trial of 628 Chinese patients type 2 diabetes receiving JADE-augmented integrate care, cardio-metabolic risk factors improved in both groups with peer support reducing hospitalization rate, especially in those with negative emotions [15]. While peer support has been shown to improve glycemic control, few studies reported its impact on healthcare utilisation [24]. In this study, we used basic stratification analyses to identify differentiating characteristics between patients who had been hospitalized and those who had not. We then applied these characteristics in a SEM to account for the confounding effects of multicollinearity to better understand factors of hospitalization. We found a consistent, independent relationship between those with improvement in negative emotions and reduced hospitalization. We also found that patients with heart or kidney disease were more likely to have reductions in hospitalization when receiving peer support. Lastly, we found an independent relationship between those with improvement in negative emotions and improvement in medication adherence.

### Peer support and its impact on comorbidity and hospitalization

Our study suggests that those with more severe comorbidities may benefit more from peer support with regards to reduced hospitalization. In patients with CKD or CVD, peer support tended to improve psychometric, self-care, and adherence measures, albeit short of significance (data not shown). There is a paucity of research that has examined the use of peer support to reduce disease burden and health care utilization in patients with diabetes and CKD or CVD [25–27]. Our integrative analysis suggests that amongst all patients, patients with CKD and/or CVD might benefit the most from peer support to address their unmet needs.

### Improving negative emotions on hospitalization and the role of adherence

Negative emotions can lead to increased health care utilisation in diabetes. In African Americans, the co-occurrence of diabetes and depression tripled the number of visits to emergency room and hospitalizations [28]. Other researchers have reported increased healthcare costs [29] and premature mortality [30] in people with diabetes and depression. Apart from depression, negative emotions such as anxiety, are more common in people with diabetes than healthy individuals [31]. Although anxiety in adolescents with diabetes is associated with a two-fold increase in hospital readmissions [32], similar findings have not been well documented in adult populations. Likewise, the roles of peer support and negative emotions in people with diabetes and their impact on health care utilisation have not been well-studied.

Our data show that improved negative emotions independently reduced hospitalizations and nonadherence, although we could not demonstrate a direct path between the latter two, likely due to small sample size. However, the independent effect of negative emotion on treatment adherence is noteworthy given the known impact of medication adherence on diabetes outcomes. Medication adherence independently predicted mortality and healthcare utilisation in a large British study of patients with type 2 diabetes receiving primary care [33]. In a survey of 2600 Chinese patients with type 2 diabetes, patients with depression and poor glycemic control reported frequent hypoglycemia, a common cause of hospitalization in diabetes, which was mediated in part by poor treatment adherence [34]. In a large study of insured Americans, improved medication adherence reduced the odds of hospitalization or visits to an emergency department by 13% after adjusting for co-morbidities and socioeconomic status [35]. By converting a non-adherent to an adherent population, the authors modelled that close to a million hospitalizations and emergency department visits could be prevented in the United States.

Although we did not show a significant interaction of peer support and negative emotions in our own SEM likely due to small sample size, the independent relationships of improved negative emotions on hospitalization reduction and improved medication adherence suggest the need for more research on these inter-relationships.

### Strengths and limitations

Strengths of this study include the collection of thorough physical, psychological and behavioural assessments in conjunction with documentation of healthcare utilisation, which are important quality measures. We also provide a detailed description of a technologically-enhanced integrated care and peer support program within a real-world setting. Regarding limitations, we found that some of the hypothesized paths, for example, effects of peer support on hospitalization through negative emotions and adherence, were not supported by the final model, which might be due to the strong confounding effect of JADE care through comprehensive assessment and education in all patients. Psychometric measurements were only measured between pre and post intervention, so changes in negative emotions could be modulated by hospitalization itself, thereby confounding the effect. We also recognized that the metabolic parameters (BP, obesity, lipids) resulted in poor fit in the SEM analysis, although univariate analysis showed that patients with or without hospitalizations had similar control of cardiometabolic risk factors. Furthermore, the greater effect size of peer support on daytime than nighttime admissions were not immediately evident. It is plausible that peer support could only alleviate hospitalizations arising from minor events which might be linked to negative emotions, but not those due to serious illness. Despite these limitations, the factor loadings, path coefficients, and model statistics of our final SEM were significant, supporting our conclusions on the relationships between peer support, comorbidities, medication adherence, and hospitalization. That said, due to the exploratory nature of this post hoc analysis and the relatively small sample size, the positive effects of a peer support intervention on hospitalization require replication and more detailed investigations.

In chronic diseases, factors other than negative emotions and medication adherence, such as ethnic minority status and incomes can also influence clinical outcomes. These parameters were not evaluated in our study. We also did not measure and adjust for comorbidities unrelated to diabetes or capture private health care utilisation. However, these unmeasured variables were expected to be equally distributed between the two groups given adequate randomization. The generalizability of this study was limited by the volunteer bias, as only 23% of eligible patients agreed to be peer supported, suggesting a more targeted approach is needed in implementing these peer support programs.

## Conclusion

In patients with type 2 diabetes receiving technologically-enhanced multidisciplinary care, reduction in negative emotions reduced hospitalization and medication nonadherence, while peer support reduced hospitalization in patients with cardiovascular-renal complications. Taken together, these findings highlight the complex inter-relationships between physical and psychological health and hospitalization, and the importance of structuring care processes so as to identify high-risk patients who might benefit from additional peer support.

## Abbreviations

CDDS-15: 15-item Chinese Diabetes Distress Scale
CKD: Chronic kidney disease as estimated glomerular filtration rate 15–60 ml/min/1.73m^2^
CVD: Cardiovascular complication including coronary heart disease (myocardial infarction, unstable angina, percutaneous coronary intervention, coronary bypass operation), stroke, peripheral vascular disease (lower extremity amputation, absent foot pulses with ankle:brachial ratio < 0.9 and lower limb revascularization)
DASS-21: 21-item Depression Anxiety Stress Scale
JADE: Joint Asia Diabetes Evaluation
Morisky Medication Adherence Scale: 4-item Medication Adherence Score
PEARL: Peer Empowerment And Remote communication Linked by information technology
PHQ-9: 9-item Patient Health Questionnaire assessing depressive symptoms
